# Application of Interrupter Resistance and Spirometry Techniques in Pediatric Pulmonary Medicine: Feasibility and Concordance in Healthy Children Under 8 Years

**DOI:** 10.3390/medicina61071265

**Published:** 2025-07-13

**Authors:** Rim Kammoun, Farah Gargouri, Asma Haddar, Halil İbrahim Ceylan, Valentina Stefanica, Walid Feki, Hatem Ghouili, Ismail Dergaa, Kaouthar Masmoudi

**Affiliations:** 1 Physiology and Functional Exploration Service, University Hospital Habib Bourguiba, Sfax 3000, Tunisia; rimkammoun@yahoo.fr (R.K.); farahgargouri8@gmail.com (F.G.); haddarasma26@gmail.com (A.H.); 2 Physical Education of Sports Teaching Department, Faculty of Sports Sciences, Atatürk University, Erzurum 25240, Türkiye; 3 Department of Physical Education and Sport, Faculty of Sciences, Physical Education and Informatics, National University of Science and Technology Politehnica Bucharest, Pitesti University Center, 110040 Pitesti, Romania; 4 Department of Respiratory Medicine, Hedi Chaker University Hospital, University of Sfax, Sfax 3029, Tunisia; fki_walid@yahoo.fr (W.F.); kaouthar.masmoudi.km@gmail.com (K.M.); 5 Research Unit: Sports Science, Health and Movement, High Institute of Sport and Physical Education of Kef, University of Jendouba, El Kef 8100, Tunisia; hatemghouili@gmail.com; 6 High Institute of Sport and Physical Education of Ksar Said, University of Manouba, Manouba 2010, Tunisia; phd.dergaa@gmail.com

**Keywords:** airway resistance, correlation, lung function, preschool, pulmonary testing, respiratory assessment, clinical evaluation, pediatric care

## Abstract

*Background and Objectives*: Pediatric pulmonary medicine relies heavily on accurate lung function assessment, yet conventional spirometry presents challenges in children due to cooperation requirements. In this context, the interrupter resistance technique (Rint), a method used in pediatric pulmonology, offers a potentially more feasible alternative for evaluating airway resistance in younger populations. This study aimed to assess the feasibility and clinical concordance between expiratory interrupter resistance (Rint(e)) and standard spirometry in healthy children under 8 years, thus contributing to the development of age-appropriate pulmonary function testing in pediatric medicine. *Materials and Methods*: A cross-sectional study was conducted on 200 healthy children (aged 2–8 years) in Tunisia. Pulmonary measurements were taken using a handheld device for both Rint(e) and spirometry. Feasibility rates were calculated, and correlations between the techniques were statistically analyzed. *Results*: Rint(e) showed significantly higher feasibility than spirometry (82.5% vs. 34.5%, *p* < 0.05). While older children had higher success rates with both techniques, feasibility was independent of sex, BMI, and passive smoking exposure. Moderate negative correlations were found between log Rint(e) and FEV1/FVC indices. *Conclusions*: In pediatric pulmonary assessment, Rint(e) demonstrated higher feasibility than spirometry among young children, making it a practical complementary method in clinical settings. However, due to only moderate correlation with spirometric indices, Rint(e) cannot yet replace spirometry in diagnostic use. Its integration into pediatric medicine may help address the gap in functional respiratory evaluation for children under the age of 8.

## 1. Introduction

Pulmonary function testing (PFT) serves as an essential tool for diagnosing and managing chronic respiratory diseases in children. These tests provide valuable information for clinical diagnosis, disease monitoring, and evaluation of therapeutic interventions [1,2]. The ability to accurately assess lung function in young children is crucial for early identification and management of respiratory conditions that may affect long-term respiratory health [1,2,3]. However, selecting the appropriate PFT method for young children remains challenging due to varying levels of required cooperation and technical considerations [1].

Conventional spirometry has long been regarded as the gold standard technique for assessing pulmonary function [3]. It effectively evaluates proximal airway function and confirms obstructive ventilatory defects (OVD). However, spirometry presents significant limitations in pediatric populations as it requires substantial patient cooperation, making it typically feasible only in children older than 5 years [4]. Younger children often struggle to perform spirometry successfully due to their inability to follow complex breathing instructions and maintain the required effort throughout the test [4]. This limitation creates a significant gap in respiratory assessment capabilities for preschool children.

Alternative techniques requiring minimal cooperation have been proposed for younger children, including the measurement of pulmonary resistance by airflow interruption (Rint), the forced oscillation technique [5], and gas wash techniques [1,6].

The Impulse Oscillometry (IOS) assesses the mechanical properties of the respiratory system by superimposing external oscillations during tidal breathing. This technique measures respiratory impedance, encompassing both resistance and reactance, across a range of frequencies. IOS provides detailed information on central and peripheral airway resistance. It is simple but requires more complex equipment that is not available in all medical centers [7,8].

The Negative Expiratory Pressure (NEP) technique evaluates flow limitation during tidal expiration by applying a brief negative pressure at the mouth. It is relatively easy to perform but is less standardized across centers and may be more affected by technical variables such as mask fit and patient posture [9].

The Rint method, particularly during expiration (Rint(e)), stands out as a non-invasive, simple test that assesses airway resistance during quiet breathing [10]. This technique involves a brief interruption of airflow at the mouth for milliseconds, during which alveolar pressure equilibrates with mouth pressure [11]. Previous studies have demonstrated significant correlations between Rint and whole-body plethysmography airway resistance [12], as well as resistance measured by the forced oscillation technique [13]. However, when compared with spirometry, Rint has not consistently demonstrated comparable sensitivity for detecting airway obstruction. Boccaccino et al. found no correlation between forced expiratory volume in the first second (FEV1) and Rint values before or after bronchodilator testing [13].

Based on these research gaps, our study aimed to compare the feasibility of expiratory Rint (Rint(e)) and spirometry in healthy Tunisian children aged 2–8 years, and to assess the relationship and agreement between the results of spirometry and Rint(e). This investigation addresses the need for validated age-appropriate pulmonary function assessment methods in this specific population.

## 2. Materials and Methods

### 2.1. Ethical Approval

This study protocol received approval from the local Ethics and Clinical Research Committee of Hospital Habib Bourguiba, Sfax, Tunisia, under the reference number CPP SUD N0076/2018. Parents or legal guardians of all participants received comprehensive information about the study aims and their rights, including the option to refuse participation or withdraw at any time. Written informed consent was obtained from all parents or legal guardians before enrollment.

### 2.2. Sample Size Calculation

Based on the results of Merkus et al. [14], we calculated the required sample size using the formula for estimating a proportion with a specified level of precision:

*n* = (Z^2^·× p ×(1 − p))/d^2^

where

- *n* is the required sample size,
- Z is the Z-value corresponding to the desired confidence level (1.96 for 95% confidence),
- p is the estimated proportion of the population (based on previous studies),
- d is the margin of error.

Using the data from Merkus et al. [14], we estimated the proportion (p) to be 0.5, which is a conservative estimate that maximizes the required sample size. The margin of error (d) was set at 0.07.

Substituting these values into the formula: n = (1.96^2^ ×·0.5·× (1 − 0.5))/0.07^2^ ≈ 196.

To account for potential dropouts and non-responses, we increased the sample size by 10%, resulting in: n_adjusted = 196 × 1.10 ≈ 216.

However, considering logistical constraints and the feasibility of recruiting participants, we aimed for a final sample size of 200 children. This sample size was chosen to ensure sufficient statistical power while maintaining practical feasibility for the study.

### 2.3. Participants

Two hundred healthy children, aged between 2 and 8 years, were recruited from three school nurseries and seven kindergartens in Sfax, Tunisia, between May 2018 and January 2020.

**Figure 1:**
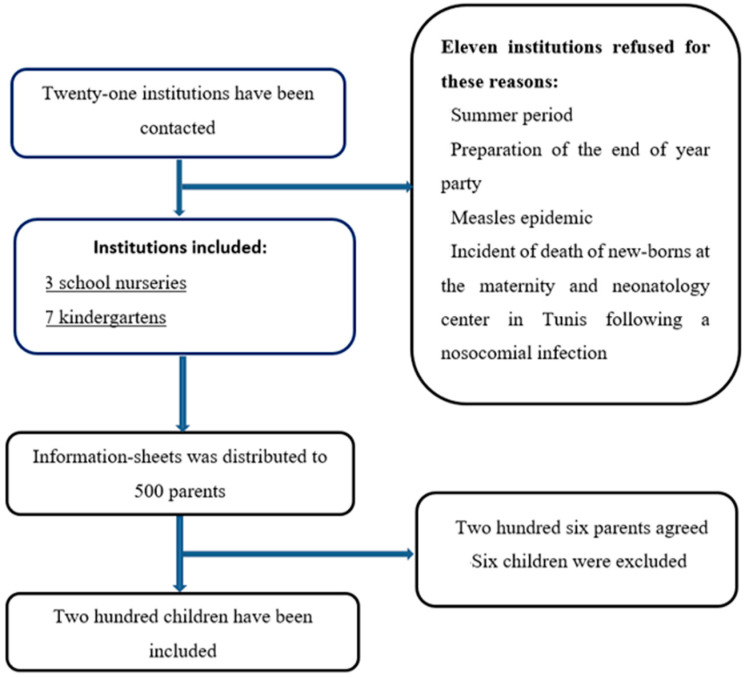
A chart illustrating the recruitment process of institutions and children.

The recruitment process consisted of two sequential phases: an initial phase at the institutional level, followed by a second phase at the participant level. Kindergartens and school nurseries located within a 10-kilometer radius of central Sfax were considered eligible. At the same time, those situated in recognized industrial areas were excluded due to elevated levels of environmental pollution that may interfere with the Rint measurement. Eligible institutions were contacted, and the study objectives, procedures, benefits, and limitations were clearly explained to their staff. Upon receiving approval, the inclusion and exclusion criteria for children were reviewed with them. Information sheets, parental consent forms, and questionnaires were then distributed by the institution staff to parents of children meeting the eligibility criteria and willing to participate in the study. Inclusion criteria comprised healthy asymptomatic children aged 2–8 years, with or without passive smoking exposure. Exclusion criteria included a history of prematurity, asthma, recurrent bronchitis, heart disease, other conditions that might interfere with lung function, or recent episodes of acute bronchitis within the past month. Once the completed and signed documents were received, the eligibility of each child was reassessed. Children or parents who decided to withdraw from the study were also excluded from the analysis. The recruitment process is illustrated in Figure 1.

### 2.4. Experimental Design

This was an observational, cross-sectional study comparing two methods of pulmonary function testing in healthy children. Demographic variables, including sex, age, and smoking exposure status, were collected. Weight (W) in kilograms (kg) and height (H) in meters (m) were measured using an electronic scale. Body mass index (BMI) was calculated by dividing weight by the square of height (in meters). Children were classified into three groups based on their BMI: non-obese, overweight, and obese, following the classification by Cole et al. [15]. The clinical and anthropometric characteristics of the study population are presented in Table 1.

### 2.5. Used Tests

#### 2.5.1. Rint(e) Measurement

The Rint(e) was measured using a handheld device (Micro Medical Limited, P.O. Box 6, Rochester, Kent, ME1 2AZ, England), interfaced with a laptop computer, before performing spirometry. Measurements were conducted with children in a sitting position, breathing quietly through a cardboard mouthpiece (28 mm diameter) with a clipped nose and a slightly extended neck. A parent or kindergarten personnel supported the child’s head to minimize the effects on upper airway compliance. During quiet breathing, an automatic occlusion was triggered during expiration at the peak of tidal flow. The valve interrupter closed within 10 ms and remained closed for 100 ms.

For acceptable measurements, the pressure–time curve needed to show a sharp pressure increase immediately following occlusion, high-frequency oscillations, and a smooth pressure increase. Measurements were considered unacceptable if the child breathed irregularly, if the curve was flat (indicating leakage), or if there were signs of upper airway compliance. Feasibility of the Rint(e) measurement was defined as the child’s ability to complete at least five technically acceptable and reproducible trials. If the child was unable to achieve this due to factors such as poor cooperation or difficulty following instructions, the measurement was considered unacceptable. Accordingly, feasibility was assessed by calculating the proportion of children who achieved acceptable Rint(e) measurements out of the total number of children who attempted the test.

**Figure 2:**
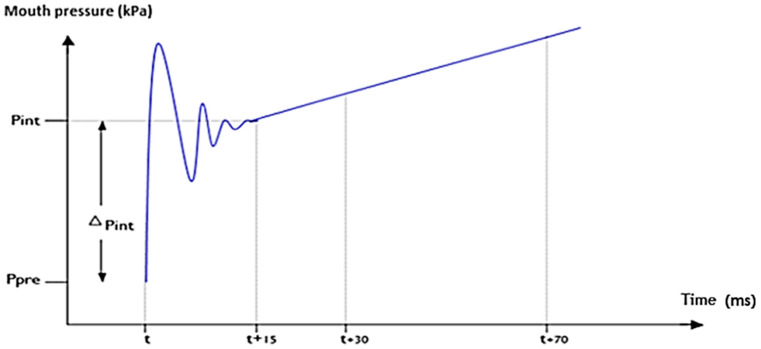
Measurement of pulmonary resistance by expiratory flow interruption by linear backwards extrapolation method at T + 15 ms (xxx).

According to the ATS/ERS guidelines of 2007 [16], Rint(e) calculation (KPa/l/s) requires measurement of airflow and alveolar pressure variation. Airflow (l/s) was measured using a flowmeter just before occlusion, and alveolar pressure (KPa) was obtained from the buccal pressure curve by backwards extrapolation to 15 ms after valve closure (T + 15 ms), as illustrated in Figure 2.

The algorithm used by the device to calculate Rint(e) was based on back-extrapolation, involving a two-point linear regression after complete valve closure. The time of complete valve closure (T) corresponded to the time occurring at 25% of the peak value of the first oscillation upstroke. Points were based on mean pressure values centered on T+30 ms and T + 70 ms, which were linearly back-extrapolated to T + 15 ms. The difference between this calculated pressure and pre-occlusion mouth pressure was divided by the flow to calculate Rint(e).

The final Rint(e) value was the median of five test values, expressed as a percentage of the predicted value established by Merkus et al. [14]. Lung function characteristics from both Rint(e) and spirometry measurements are presented in Table 2.

#### 2.5.2. Spirometry

All children performed flow–volume spirometric tests using a handheld spirometer (Micro Medical Limited, superspiro, Rochester, Kent, UK) in a sitting position. At least three acceptable tests were obtained in accordance with ATS/ERS recommendations [17]. The highest values of forced expiratory volume in the first second (FEV1 (L/s)), Forced Vital Capacity (FVC (L)), FEV1/FVC ratio, Peak Expiratory Flow (PEF (L/s)), and Maximum Median Expiratory Flow (MMEF (L/s)) were selected as the representative values. An obstructive ventilatory defect (OVD) was defined as a reduced FEV1/FVC ratio below the lower limit of normal, according to the Global Lung Initiative reference (GLI 2012) [17,18].

### 2.6. Statistical Analysis

Statistical analysis was performed using SPSS version 20 software. The Kolmogorov–Smirnov test was used to assess the normal distribution of quantitative variables. Quantitative data (age, height, weight, BMI, FEV1, FVC, PEF, FEV1/FVC, MMEF, Rint(e)) were reported as mean ± standard deviation or as median (1st quartile, 3rd quartile). Categorical variables (sex, physical stature, presence of proximal OVD, and parental smoking exposure) were expressed as percentages. Relationships between Rint(e) measurement and categorical variables were assessed using ANOVA or Mann–Whitney tests. Spearman’s and Pearson’s tests examined correlations between measured Rint(e) values and other quantitative variables. A *p*-value < 0.05 was considered significant. The linearity of association between Rint(e) and quantitative measures was verified graphically. Logarithmic transformation was applied to preserve the normality of the distribution of the Rint(e) measurement. Factors determining test feasibility are summarized in Table 3.

Agreement between the two techniques (Rint(e) and spirometry) was assessed using Cohen’s Kappa test. A Cohen’s Kappa coefficient between zero and 0.2 corresponds to poor agreement, while a coefficient between 0.81 and 1 corresponds to almost perfect agreement.

## 3. Results

### 3.1. Characteristics of the Study Population

Of the 500 parents who received study information, 206 parents agreed to their children’s participation. On the study day, three children were sick and three had a history of asthma, resulting in 200 children included in the final analysis. The median age was 5 years (range: 2.38–8.15 years), with 33.5% of participants older than 6 years. The study included equal numbers of boys and girls (100 each). Fifty-three children (41%) were exposed to passive smoking. Regarding BMI classification, 70% had a normal BMI, 16% were overweight, and 14% were obese. Table 1 summarizes the clinical and anthropometric characteristics of the study population.

### 3.2. Lung Function Characteristics

All children who underwent successful spirometry testing had normal results without obstructive ventilatory defects. The median Rint(e) value was 0.64 KPa/L/s (range: 0.15–1.37), corresponding to 82.32% (range: 26.69–252.83%) of predicted values according to Merkus’ 2001 equations [14]. A total of 7.9% (13 children) had a Rint(e) measurement above the upper limit of normal [14] despite the normal spirometry.

For spirometry, the mean FEV1 was 1.20 ± 0.32 L/s (87.92 ± 12.93% of predicted), the mean FVC was 1.31 ± 0.38 L (86.85 ± 14.50% of predicted), and the mean FEV1/FVC ratio was 0.90 ± 0.04. Table 2 presents the complete results of pulmonary function testing.

FEV1 (%): the measured FEV1 value expressed as a percentage of the predicted value established from the GLI 2012 equations. FVC (%): the measured value of FVC expressed as a percentage of the expected value established from the GLI 2012 equations. Rint(e) (%): the value of the measured Rint(e) expressed as a percentage of the predicted value established from the Merkus equations [14].

### 3.3. Feasibility of Pulmonary Function Tests

The feasibility of Rint(e) measurement was significantly higher than spirometry (82.5% [N = 165] versus 34.5% [N = 69], *p* < 0.05). Cohen’s kappa coefficient indicated poor agreement between the feasibility of the two techniques (0.118, *p* < 0.05). Sixty-four children (38.8%) with successful Rint(e) had acceptable spirometry, while 14.2% of children with successful spirometry had unacceptable Rint(e).

Furthermore, when comparing children who successfully performed both Rint(e) and spirometry (n = 64) with those who completed only Rint(e) but failed spirometry (n = 101), age emerged as the only significant distinguishing factor. Children with both acceptable Rint(e) and spirometry results were significantly older than those with acceptable Rint(e) alone (6.22 ± 1.16 years vs. 5.04 ± 1.20 years; *p* = 0.00).

Gender, BMI status, and passive smoking exposure did not significantly affect the feasibility of spirometry. Table 3 summarizes the relationships between these factors and test feasibility.

The chi-square test was used to compare the feasibility rates of the Rint(e) measure and spirometry between the two sexes, between the two groups of children exposed and not exposed to passive smoking, and between children aged above or below six years. The Kruskal–Wallis test was used to compare the feasibility of measuring Rint(e) and spirometry between the three groups of children with obesity, overweight, and correct BMI. Rint(e) feasibility is calculated as the proportion of children who achieved acceptable Rint(e) measurements out of the total number of children who attempted the test. *p* < 0.05: significant, N: number, (N): number of patients.

### 3.4. Relationship Between Rint(e) and Spirometry Values

**Figure 3:**
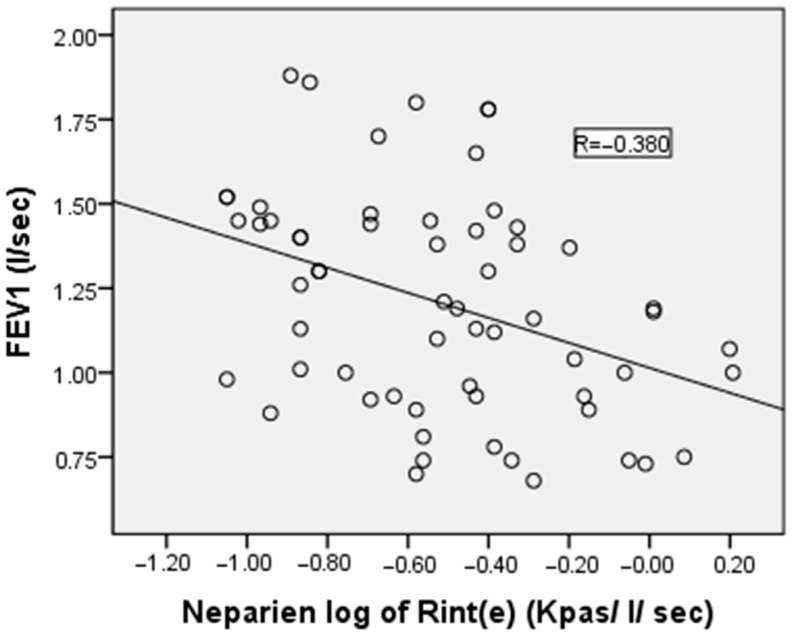
Correlation between the airflow interruption method’s log of pulmonary resistance by expiratory flow interruption and forced expiratory volume in the first second of the healthy children in our study. R represents the value of the Pearson correlation coefficient, which is used to note the strength and direction of the relationship between variables.

Pearson’s correlation analysis revealed no significant correlation between logarithmically transformed Rint(e) values and both FEV1/FVC ratio and MMEF. However, moderate negative correlations were observed between log Rint(e) and FEV1 and FVC, with correlation coefficients of −0.380 and −0.406, respectively (*p* < 0.05 for both). The correlation between the measured value of Rint(e) and FEV1 is illustrated in Figure 3.

## 4. Discussion

Our study investigated the feasibility and correlation of two pulmonary function testing methods in healthy Tunisian children below 8 years of age. The primary findings demonstrated significantly higher feasibility of the interrupter resistance technique during expiration compared to conventional spirometry, with moderate correlations between these methods.

In our study, the overall feasibility rate was 82.5% for Rint(e) and 34.5% for spirometry, demonstrating that Rint(e) is considerably easier in young children. This substantial difference highlights the practical challenges of performing spirometry in this age group, likely due to the need for sustained expiratory effort, which is difficult for children with small lung volumes and a lack of cooperation.

Our findings are consistent with those reported by Tatar and Man [19], who assessed the feasibility of Rint in children with acute asthma. In their study, which included 66 children aged from 2 to 6 years, 90% were able to successfully perform Rint measurements, whereas only 27% achieved acceptable spirometry. While their population consisted of acutely ill asthmatic children, and ours included healthy children, the conclusion remains similar: spirometry is more challenging to perform in preschool-aged children, regardless of clinical status [19,20]. On the other hand, the Rint technique is more straightforward to accomplish in children, irrespective of their clinical status. This finding is further supported by the study of Arets et al., which demonstrated that the feasibility of Rint was comparable between healthy and asthmatic children, with success rates of 92% and 91%, respectively [21].

These findings reinforce the value of alternative lung function techniques, such as Rint(e), especially for use in preschool children for whom standard spirometry may not be feasible.

Interestingly, some studies have reported higher spirometry success rates in young children, ranging from 77% to 90% [22,23]. These discrepancies may be explained by methodological differences, including variations in participant numbers and acceptability criteria [23], the use of FEV0.5 or FEV0.75 s indices instead of FEV1 [20,24], and the implementation of incentive methods [22]. For instance, Nystad demonstrated that requiring only two acceptable flow–volume curves instead of three increased success rates from 68% to 92% [24]. Similarly, the Aurora study showed that using FEV0.5 instead of FEV1 improved success rates from 58% to 75% in children aged 2–5 years [25].

Our analysis of factors affecting test feasibility revealed that age was the only significant determinant, with both techniques more successful in children older than 6 years. This finding supports previous observations by Zuriarrain Reyna et al., who reported Rint(e) acceptability of approximately 72.6% in children under 7 years [11]. While Rint(e) is generally easier to perform, its success rate was still notably lower in children under 6 years, suggesting that even this technique requires a minimum level of cooperation that younger children may struggle to provide.

Regarding the relationship between the two techniques, we found moderate negative correlations between logarithmically transformed Rint(e) values and both FEV1 and FVC (r = −0.380 and −0.406, respectively). This finding aligns with previous studies, which have shown moderate to good, but not perfect, correlations between these parameters [19,26,27,28]. The incomplete correlation may be explained by physiological differences in what each technique measures: Rint(e) evaluates not only proximal airway resistance but also lung tissue and chest wall resistance [29], providing a more comprehensive assessment of total respiratory system resistance. Additionally, the use of absolute values in the correlation analysis may have limited the strength of the correlation. Expressing both the Rint and spirometric parameters as Z-scores could potentially enhance the correlation by accounting for age, height, and weight. This may provide a more standardized comparison between the two techniques, thereby improving the accuracy of data interpretation [30].

Oswald demonstrated that Rint(e) was inversely correlated with obstruction severity, as estimated by the FEV1/FVC ratio [31]. At the same time, Beydon et al. found correlations between Rint(e) and both FEV1 and FVC in asthmatic children before and after bronchodilator therapy [10].

All children who successfully performed spirometry in our study had normal results without obstructive patterns. This prevented us from evaluating the sensitivity of Rint(e) for detecting airway obstruction. Previous research has indicated limited sensitivity of Rint(e) for this purpose, with Beydon et al. reporting sensitivity of only 42% [10], similar to findings by Delacourt et al. (33%) [27] and Mekenze et al. (60%) [32]. A likely explanation is that in patients with airway obstruction, the brief interruption time may be insufficient for mouth and alveolar pressures to equilibrate, resulting in underestimated resistance values.

In addition to Rint(e), as an alternative to spirometry, other techniques such as IOS and the NEP method have been widely performed in young children due to their simplicity, minimal cooperation requirements, and reproducibility of results. IOS measures respiratory system impedance by applying pressure oscillations during tidal breathing, offering detailed information on both central and peripheral airway resistance. NEP, on the other hand, assesses flow limitation by applying negative pressure at the mouth during expiration [9].

Compared to spirometry and to Rint(e), IOS provides a broader frequency spectrum of respiratory mechanics, potentially enhancing sensitivity to peripheral airway changes [7,8]. However, it requires specialized equipment.

## 5. Limitations

Our study has several limitations that should be considered when interpreting the results. First, we included only healthy children without respiratory conditions, which prevented an assessment of how these techniques perform in diagnosing or monitoring respiratory diseases. Second, bronchodilator testing was not performed, limiting our ability to evaluate the techniques’ responsiveness to airway changes. Third, although our sample size of 200 children was larger than that of many previous studies, subgroup analyses based on age had reduced statistical power, particularly for the younger age groups, where feasibility was lowest. Fourth, we used only one type of interrupter device and analysis algorithm, so that results might differ with other equipment or analytical approaches. Finally, our cross-sectional design provides no information about the longitudinal reliability of these measurements or their predictive value for respiratory health outcomes.

## 6. Conclusions

Our study demonstrates that Rint(e) is significantly more feasible than spirometry in healthy Tunisian children under 8 years of age, with age being the key factor influencing test success. However, due to its weak to moderate correlation with spirometric indices and limited sensitivity for detecting airway obstruction, Rint(e) should not be considered a direct alternative. Its interpretation must account for potential upper airway influences and its limited ability to distinguish between central and peripheral airway obstruction. These limitations are particularly relevant in heterogeneous patient populations or when subtle abnormalities have significant clinical implications. Future studies incorporating bronchodilator testing are needed to define better the clinical utility of Rint(e) in pediatric respiratory assessment.

## 7. Future Directions and Applications

The Rint is a simple test that can be easily performed by children, regardless of their clinical status. Clinically, the Rint has several critical applications. It can be used for the early assessment of pulmonary function, particularly in settings where simple spirometry may be challenging. Furthermore, it serves as a helpful adjunct in the diagnosis of wheezy children or children presenting with nonspecific respiratory symptoms. By assessing bronchodilator response and/or conducting bronchial challenge tests, the Rint can aid in identifying airway reversibility or hyperresponsiveness, supporting differential diagnosis. Additionally, the Rint is well-suited for use in therapeutic intervention trials, where it can serve as a sensitive outcome measure to evaluate the efficacy of respiratory treatments in pediatric populations. The use of standardized parameters, such as z-scores, in future studies would be of interest, as they may enhance the accuracy of data interpretation and improve the strength of statistical analyses.

## Figures and Tables

**Table 1:** Clinical and anthropometric characteristics of the healthy children in our study.

	Total Sample	Girls	Boys
N	200	100	100
Age (years)	5.1 (2.38–8.15)	5.00 (2.81–8.00)	5.08 (2.38–8.00)
Child aged > 6 years	33.5%	32%	35%
BMI (kg/m^2^)	16.54 (12.68–27.66)	16.34 (13.08–26.23)	16.87 (12.68–27.66)
Weight (kg)	21.30 (9.60–45.00)	21.10 (13.40–39.10)	21.90 (9.60–45.00)
Height (cm)	113.50 (80.00–143.00)	113.00 (80.00–143.00)	114.62 (80.00–136.00)
Exposure to passive smoking	41% (82)	48% (48)	34% (34)
Physical stature			
Obesity	14% (28)	12% (12)	16% (16)
Overweight	16% (32)	15% (15)	17% (17)
Correct BMI	70% (140)	73% (73)	67% (67)

N: number of patients; the results are expressed by the median (minimum–maximum); the qualitative variables expressed in percentage (%); BMI: body mass index. Obesity and overweight have been defined according to the Cole definition. N: number, (N): number of patients.

**Table 2:** Spirometric and pulmonary resistance by flow interruption data of the healthy children in our study.

Settings	Values
Rint(e) Data (N = 165)	
Rint(e) (Kpa/L/s)	0.64 (0.15–1.37)
Rint(e) (%)	82.32 (26.69–252.83)
Spirometric data (N = 69)	
FEV1 (L/s)	1.20 ± 0.32
FEV1 (%)	87.92 ± 12.93
FVC (L)	1.31 ± 0.38
FVC (%)	86.85 ± 14.50
FEV1/FVC	0.90 ± 0.04
MMEF (L/s)	1.56 ± 0.41
FET (s)	2.16 ± 0.80

N: number of patients; s: second; the results of Rint(e) are expressed by the median (minimum–maximum); the results of FEV1, FVC, MMEF, and TEF are expressed by the mean ± standard deviation; Rint(e): pulmonary resistance by expiratory flow interruption; FEV1: forced expiratory volume in the first second; FVC: forced vital capacity; FEV1/FVC: FEV1/FVC ratio; MMEF: Maximum Median Expiratory Flow; FET: forced expiration time.

**Table 3:** Factors determining the feasibility of performing the airflow interruption method and spirometry of the healthy children in our study.

	Rint(e) Feasibility (%) (N)	*p*	Spirometry Feasibility (%) (N)	*p*
Age		**0.007**		**0.000**
>6 ans (67)	94% (63)		55% (37)	
<6 ans (133)	77% (102)		24% (32)	
Gender		0.802		0.963
Boys (100)	83% (83)		35% (35)	
Girls (100)	82% (82)		34% (34)	
Passive smoking		**0.00**		**0.605**
Exposed (82)	94% (77)		37% (30)	
Unexposed (118)	75% (88)		33% (39)	
Physical status		0.918		0.302
Obesity (28)	86% (24)		47% (13)	
Overweight (32)	87% (26)		28% (9)	
Correct BMI (140)	80% (115)		34% (47)	

The chi-square test was used to compare the feasibility rates of the Rint(e) measure and spirometry between the two sexes, between the two groups of children exposed and not exposed to passive smoking, and between children aged above or below six years. The Kruskal-Wallis test was used to compare the feasibility of measuring Rint(e) and spirometry between the three groups of children with obesity, overweight, and correct BMI. Rint(e) feasibility is calculated as the proportion of children who achieved acceptable Rint(e) measurements out of the total number of children who attempted the test. *p* < 0.05: significant, N: number, (N): number of patients. Statistically significant results are indicated in bold.

## Data Availability

The raw data supporting the conclusions of this article will be made available by the authors on request.
